# KarMMa-RW: comparison of idecabtagene vicleucel with real-world outcomes in relapsed and refractory multiple myeloma

**DOI:** 10.1038/s41408-021-00507-2

**Published:** 2021-06-18

**Authors:** Sundar Jagannath, Yi Lin, Hartmut Goldschmidt, Donna Reece, Ajay Nooka, Alicia Senin, Paula Rodriguez-Otero, Ray Powles, Kosei Matsue, Nina Shah, Larry D. Anderson, Matthew Streetly, Kimberly Wilson, Hoa Van Le, Arlene S. Swern, Amit Agarwal, David S. Siegel

**Affiliations:** 1 Mount Sinai Hospital, New York, NY USA; 2 Mayo Clinic, Rochester, MN USA; 3 University Hospital Heidelberg, Internal Medicine V and National Center for Tumor Diseases (NCT), Heidelberg, Germany; 4 Princess Margaret Cancer Centre, Toronto, ON Canada; 5 Emory University School of Medicine, Atlanta, GA USA; 6 Institut Català d’Oncologia, Badalona, Barcelona, Spain; 7 Clínica Universidad de Navarra, Pamplona, Spain; 8 Cancer Centre London, London, UK; 9 Kameda Medical Center, Kameda-honchō, Japan; 10 University of California San Francisco, San Francisco, CA USA; 11 Simmons Comprehensive Cancer Center, UT Southwestern Medical Center, Dallas, TX USA; 12 Guy’s and St Thomas’s Hospital, London, UK; 13 Bristol Myers Squibb, Princeton, NJ USA; 14 Hackensack University Medical Center, Hackensack, NJ USA

**Keywords:** Cancer, Medical research

## Abstract

Patients with relapsed and refractory multiple myeloma (RRMM) who are triple-class exposed (to an immunomodulatory agent, proteasome inhibitor, and anti-CD38 antibody) have limited treatment options and there is no standard of care. Idecabtagene vicleucel (ide-cel, bb2121), a BCMA-directed CAR T-cell therapy, demonstrated efficacy in triple-class exposed RRMM patients in the KarMMa trial (NCT03361748). In this retrospective study (KarMMa-RW), patient-level data from triple-class exposed RRMM patients were merged into a single data model and compared with KarMMa using trimmed stabilized inverse probability of treatment weighting. Endpoints included overall response rate (ORR; primary), rate of very good partial response or better (≥VGPR), progression-free survival (PFS), and overall survival (OS). Of 1949 real-world triple-class exposed RRMM patients, 190 received subsequent (index) line of therapy and met KarMMa eligibility criteria (Eligible RRMM cohort). With a median follow-up of 13.3 months in KarMMa and 10.2 months in Eligible RRMM, ORR, and ≥VGPR were significantly improved in KarMMa versus Eligible RRMM (ORR, 76.4% vs 32.2%; ≥VGPR, 57.9% vs 13.7%; both *P* < 0.0001) as were PFS (11.6 vs 3.5 months; *P* = 0.0004) and OS (20.2 vs 14.7 months; *P* = 0.0006). This study demonstrated that ide-cel significantly improved responses and survival compared with currently available therapies in triple-class exposed RRMM.

## Introduction

Multiple myeloma is the second most common hematologic malignancy, with ~160,000 newly diagnosed cases and 106,000 deaths worldwide in 2018 [1]. Over the past decade, advances in treatment of multiple myeloma have resulted in a significant improvement in overall survival (OS) [2–4]. This improvement has been primarily driven by more effective combination therapies of immunomodulatory agents, proteasome inhibitors (PIs), and dexamethasone coupled with consolidation using autologous stem cell transplant [3, 5]. New treatment options for multiple myeloma are rapidly evolving, with the approval of anti-CD38 antibodies such as daratumumab and isatuximab, further improving outcomes [6–9]. Additional novel agents, such as the histone deacetylase inhibitor panobinostat, the anti-SLAMF-7 antibody elotuzumab, and the nuclear export inhibitor selinexor, have been approved by the Food and Drug Administration in recent years for use in combination regimens [10–13]. Despite dramatic progress in treatment, multiple myeloma remains largely incurable, and almost all patients eventually relapse, with worsening prognosis and survival at each relapse regardless of subsequent treatment [3, 14, 15]. Previous retrospective data for patients with multiple myeloma refractory to immunomodulatory agents, PIs, and anti-CD38 monoclonal antibodies from 14 different US academic institutions reported a median progression-free survival (PFS) of only 3−4 months and a median OS of 8−9 months [16]. Such triple-class exposed patients with relapsed and refractory multiple myeloma (RRMM) have few treatment options, even with the newly approved therapy belantamab mafodotin [17, 18], and there is no clear consensus on the optimal therapy or standard of care [16, 18–22].

Idecabtagene vicleucel (ide-cel, bb2121) is a B-cell maturation antigen (BCMA)-directed chimeric antigen receptor (CAR) T-cell therapy that has demonstrated promising safety and efficacy in RRMM. Ide-cel is generated from autologous T cells transduced with a third-generation lentiviral vector encoding a CAR specific for human BCMA, which consists of the targeting domain of anti-BCMA, costimulatory domain of 4-1BB, and T-cell activation domain of CD3ζ [23]. The phase 1 CRB-401 study (NCT02658929) reported a confirmed ORR of 76% with ide-cel, including complete responses in 39% of patients with ≥3 prior lines of therapy (including immunomodulatory agents and PIs), or double-class refractory disease, and a median PFS of 8.8 months [24]. Frequent, deep and durable responses with ide-cel were recently reported in patients who were triple-class exposed and refractory to their last regimen in the ongoing pivotal phase 2 KarMMa study (NCT03361748) [25]. The safety profile of ide-cel was consistent across both the phase 1 and 2 studies, with mostly grade 1 or 2 adverse events of cytopenia, cytokine-release syndrome, and neurotoxicity.

Large-scale, patient-level retrospective studies can provide a better understanding of outcomes with currently available therapies and help to establish benchmarks for future clinical trials [21, 22, 26, 27]. However, patient-level data in triple-class exposed RRMM patients are not well characterized, and the limited data that are available vary across geographies. Here we describe the demographics, disease characteristics, treatment patterns, and clinical outcomes in real-world RRMM patients with characteristics similar to the KarMMa study population. We also compare clinical outcomes from the real-world RRMM patients treated with currently available therapies and the patients treated with ide-cel in the KarMMa study.

## Materials and methods

### Study design and patients

**Fig. 1:**
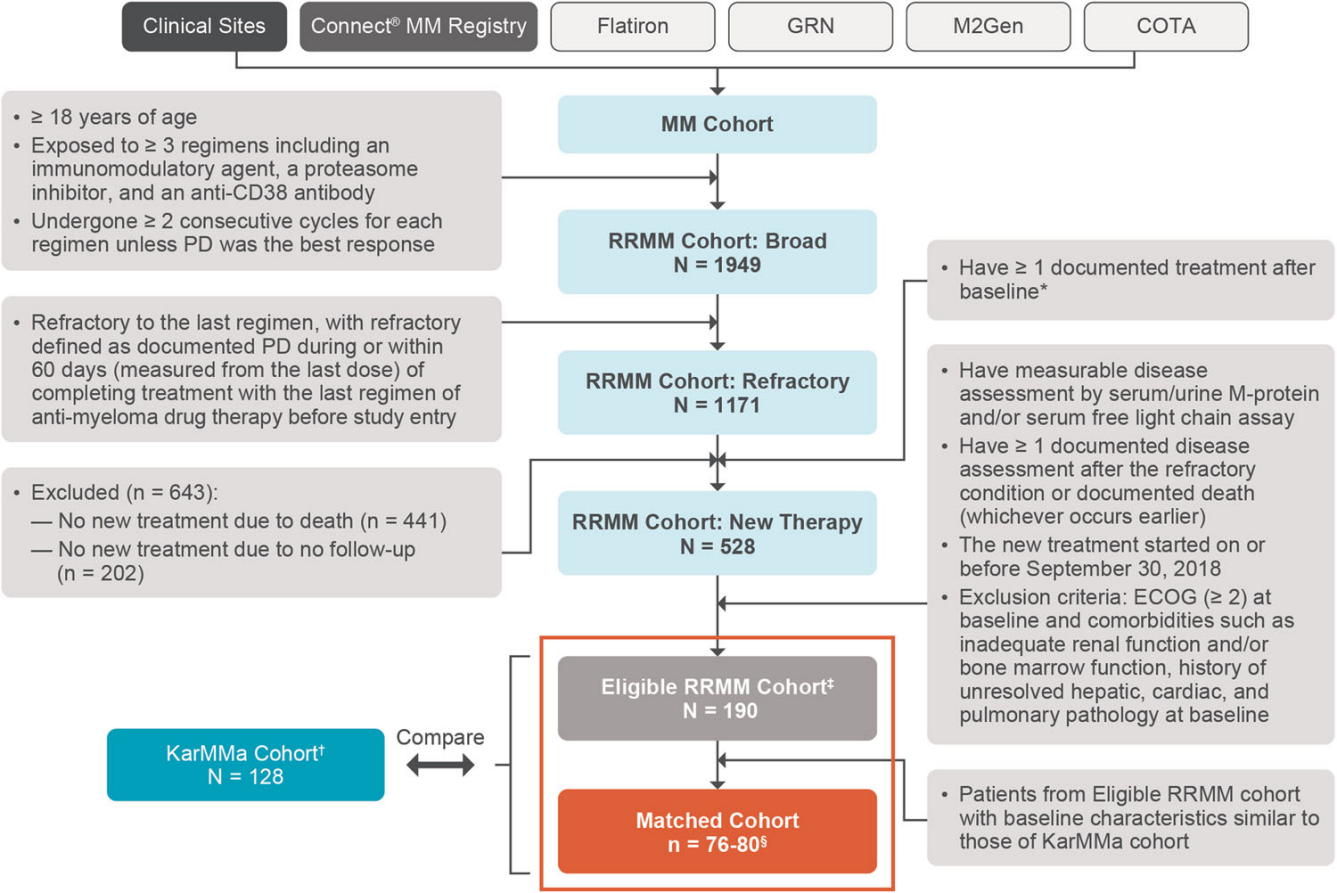
Selection process for real-world Eligible Cohort. Collection of patient-level data from clinical sites, the Connect MM Registry, and external research databases, and a description of the selection process for the Eligible RRMM cohort in the KarMMa-RW study. *Baseline was defined as when patients became refractory to their last regimen. ^†^Across all ide-cel target doses in the KarMMa study (ClinicalTrials.gov: NCT03361748); at the data cutoff of 30 October 2019, 58 patients (45.3%) had discontinued from the study, 31 (24.2%) due to death, 26 (20.3%) due to study withdrawal, and 1 (0.8%) lost to follow-up. ^‡^Overall, 108 patients (56.8%) discontinued from the study, all due to death. ^§^Numbers (ranges) of matched patients from 30 imputed datasets. COTA denotes the COTA real-world evidence database, ECOG Eastern Cooperative Oncology Group, GRN Guardian Research Network, RRMM relapsed and refractory multiple myeloma.

In this global, noninterventional, retrospective study (KarMMa-RW), and real-world patient-level data were collected from multiple sources and merged into a single data model. Real-world patients with RRMM were initially selected based on broad inclusion and exclusion criteria (Fig. 1). Patients aged ≥18 years with a documented diagnosis of multiple myeloma, who had received ≥3 prior regimens, including an immunomodulatory agent, a PI, and an anti-CD38 antibody (received on or after 16 November 2015, the earliest approval of daratumumab by the Food and Drug Administration) were included in the broad RRMM cohort. Patients in this cohort had to have received ≥3 prior regimens by 30 September 2018, to ensure sufficient follow-up; induction with or without hematopoietic stem cell transplant and with or without maintenance therapy was considered a single regimen. Additional eligibility criteria for this cohort included ≥2 consecutive treatment cycles for each regimen, unless progressive disease was the best response for that regimen. Patients exposed to any BCMA-directed therapy or gene-modified therapy were excluded.

From the broad RRMM cohort, patients who were refractory to their last antimyeloma regimen (defined as documented progression during or within 60 days of last dose before study entry) were selected for the refractory RRMM cohort (Fig. 1). Further selection yielded a subset of patients who received ≥1 documented treatment after they became refractory to their last regimen.

The Eligible RRMM cohort was selected based on additional inclusion and exclusion criteria used for the KarMMa study and International Myeloma Working Group (IMWG) response and progression criteria [28]. Patients in the Eligible RRMM cohort were refractory to their last regimen, had measurable disease based on monoclonal-protein and/or serum free light chain levels, and ≥1 documented disease assessment after the refractory condition or documented death. Additional eligibility criteria are included in Supplementary Methods.

A subset of patients from the Eligible RRMM cohort with baseline characteristics more closely matching those of patients in the KarMMa study (Matched RRMM cohort) were selected from the real-world patient-level data model and compared with the matched KarMMa cohort. Clinical outcomes in the real-world cohorts were compared with results in the KarMMa population using a composite across the target ide-cel dose levels of 150–450 × 10^6^ CAR + T cells as well as the highest target dose level of 450 × 10^6^ CAR + T cells.

### Data sources

Data were collected on RRMM patients using a set of parameters (Fig. 1). Real-world data from patients in North America and Europe were obtained from three types of data sources: clinical sites, the Connect® MM Registry [29], and external research databases. Data collection was retrospective and did not change clinical practice or patient visit schedules; each data source was in compliance with applicable national and local ethical, legal, and privacy regulations.

Clinical sites in North America and Europe were selected based on predefined criteria including number of patients treated for RRMM, ability to share patient-level data, availability of key clinical information, including current treatments and ongoing disease assessments, and correspondence of time required for data access and contracting to study timelines. More than 30 clinical sites were approached; however, several factors such as data privacy issues and contracting delays, made data collection more challenging in Europe.

Real-world data were also collected from the Connect® MM Registry (NCT01081028), a multicenter, prospective observational cohort designed to explore the natural history and real-world management of patients with newly diagnosed multiple myeloma. The Registry currently follows 3011 patients from ~250 sites in the United States.

External research databases were an enhanced collection of longitudinal data and de-identified patient-level electronic health records in the United States. Additional details can be found in Supplementary Methods.

### Data integration

Data from all sources were reformatted into the standardized study data model. Program and mapping specifications for variable transformations and derivations were created for each data element within each data source. Variable transformations included creation of common data types, formats, taxonomy, ontology, as well as data structure. Variable derivations were implemented to define a consistent definition for regimen, baseline, index date (study day 1), study entry, outcome response, comorbidities, and imputation of missing date values across all data sources. All transformations and analytic derivation decisions and their lineages to the source data were documented.

### Endpoints and assessments

The primary endpoint was ORR, defined as partial response (PR) or better per IMWG criteria [28]. Secondary endpoints included complete response (CR) rate, rate of very good partial response or better (≥VGPR), PFS, OS, duration of response (DOR), and time to response (TTR). Subgroup assessments of ORR and PFS stratified by sex, age, double-refractory status (immunomodulatory agents and PIs), and number of prior antimyeloma regimens per year were performed.

### Statistical analysis

Propensity score balancing was used to summarize the impact of covariates on treatment selection into scalar values. These values were then used for weighting individual patients in both the Eligible RRMM cohort and the KarMMa cohort, using inverse probability treatment weighting (IPTW). Furthermore, a subset of the two cohorts were matched in order to improve the balance of covariates between groups [30–32]. Steps utilized in the propensity score balancing process are reported in Supplementary Methods. Using this methodology, results in the KarMMa cohort were adjusted to account for differences in patient characteristics between cohorts and are therefore slightly different from results reported with the KarMMa study.

For primary analyses, trimmed stabilized IPTW was used to compare clinical outcomes/endpoints of interest. Baseline prognostic variables considered for IPTW were selected and ranked by a scientific steering committee. Multiple imputation was used to address missing values with propensity scores generated in each of 30 separate datasets.

The initial study protocol plan was to have variable ratio matching using the propensity score modeling to achieve an overall 2:1 ratio of the KarMMa cohort to Eligible RRMM cohort; however, due to the resulting small sample size, a matched-pair comparison between the KarMMa cohort and the Matched RRMM cohort was included as a supporting sensitivity analysis instead. An untrimmed stabilized IPTW was used to compare the KarMMa and RRMM cohorts as an additional sensitivity analysis.

Poisson regression was used to analyze ORR and ≥VGPR rates due to nonconvergence of the binomial regression, and Cox proportional hazards models were used to analyze PFS, OS, and DOR. Because analyzing CR requires a bone marrow biopsy per IMWG criteria, which is generally not available for real-world data, analyses were summarized for ≥VGPR rate to avoid underestimating depth of response in the real-world setting. All models were adjusted for unbalanced covariates and overall summary estimates generated from the 30 separate datasets.

Analyses were performed using SAS version 9.4. Descriptive analyses were performed to gain an understanding of the quality of the data and statistical distributions of characteristics of the real-world patients. Continuous variables were described by mean with standard deviation and 95% confidence interval (CI), median, upper and lower quartiles and range values. Categorical variables were reported as number and percentage with 95% CIs. Two-sided *P* < 0.05 was considered statistically significant without multiplicity adjustment.

## Results

### Patients

Patient-level data were collected from 1949 real-world patients with RRMM who received ≥3 prior regimens, including an immunomodulatory agent, a PI, and an anti-CD38 antibody. Of these 1949 real-world triple-class exposed RRMM patients, 1171 were refractory to their last regimen at baseline (RRMM cohort). The median age of patients in the RRMM cohort was 68 years, the median number of prior regimens was 5, and 19.0% of patients had received ≥7 prior regimens; 40.9% were triple-class refractory (Table 1). Among the 1171 patients in the RRMM cohort, 528 had received a subsequent line of therapy (hereafter referred to as the subsequent [index] line therapy); 643 patients were excluded for having no new treatment or due to death (*n* = 441) and lack of follow-up (*n* = 202). Additional eligibility criteria from the KarMMa study [33] were applied to the 528 patients, resulting in the selection of 190 patients for the Eligible RRMM cohort. A total of 128 ide-cel treated patients from the KarMMa study were compared with the Eligible RRMM cohort (Fig. 1). At the data cutoff (30 October 2019), 108 patients (57%) in the Eligible RRMM cohort had died.

**Table 1:** Baseline demographics and disease characteristics (RRMM, Eligible RRMM, KarMMa).

Characteristic^a^	KarMMa cohort^b^ (*N* = 128)	RRMM cohort (*N* = 1171)	Eligible RRMM cohort (*N* = 190)
Median age, years (range)	60.5 (33.0–78.0)	68.0 (32.0–95.0)	64.0 (35.0–91.0)
Male, *n* (%)	76 (59.4)	639 (54.6)	111 (58.4)
Median time since initial diagnosis, years (range)	6.0 (1.0–17.9)	4.3 (0.4–28.3)	4.2 (0.4–17.7)
ECOG performance status, *n* (%)			
0	57 (44.5)	134 (11.4)	29 (15.3)
1	68 (53.1)	328 (28.0)	72 (37.9)
2	3 (2.3)^c^	126 (10.8)	0
3	0	28 (2.4)	0
4	0	11 (0.9)	0
Missing	0	544 (46.5)	89 (46.8)
R-ISS disease stage, *n* (%)^d,e^			
I	14 (10.9)	2 (0.2)	0
II	90 (70.3)	174 (14.9)	50 (26.3)
III	21 (16.4)	37 (3.2)	7 (3.7)
Unknown	3 (2.3)	958 (81.8)	133 (70.0)
Cytogenetic abnormalities, *n* (%)			
High risk	45 (35.2)	352 (30.0)	57 (30.1)
Non-high risk	66 (51.6)	165 (14.1)	24 (12.6)
Not evaluable/missing	17 (13.3)	654 (55.8)	109 (57.4)
Presence of any plasmacytoma, *n* (%)	50 (39.1)	143 (12.2)	21 (11.1)
Median number of prior antimyeloma regimens (range)	6.0 (3.0–16.0)	5.0 (3.0–13.0)	5.0 (3.0–12.0)
Prior antimyeloma regimens, *n* (%)			
3	15 (11.7)	292 (24.9)	44 (23.2)
4	19 (14.8)	287 (24.5)	43 (22.6)
5	22 (17.2)	243 (20.8)	45 (23.7)
6	23 (18.0)	126 (10.8)	21 (11.1)
≥7	49 (38.3)	223 (19.0)	37 (19.5)
Number of prior antimyeloma regimens per year since diagnosis, *n* (%)^c^			
≤1	71 (55.5)	260 (22.2)	89 (46.8)
>1	57 (44.5)	268 (22.9)	101 (53.2)
Missing	0	643 (54.9)	0
Prior stem cell transplantation, *n* (%)			
1	76 (59.4)	525 (44.8)	101 (53.2)
>1	44 (34.4)	154 (13.2)	33 (17.4)
Prior relapse/refractory status, *n* (%)			
Immunomodulatory agent	126 (98.4)	834 (71.2)	142 (74.7)
Proteasome inhibitor	116 (90.6)	746 (63.7)	122 (64.2)
Anti-CD38 antibody	120 (93.8)	956 (81.6)	162 (85.3)
Immunomodulatory agent and proteasome inhibitor (double refractory)	114 (89.1)	580 (49.5)	102 (53.7)
Immunomodulatory agent, proteasome inhibitor, and anti-CD38 antibody (triple refractory)	108 (84.4)	479 (40.9)	82 (43.2)

*ECOG* Eastern Cooperative Oncology Group, *R-ISS* Revised International Staging System.

^a^Baseline measurements for the KarMMa study were performed within 72 h prior to lymphodepleting chemotherapy before the start of ide-cel infusion. Baseline for the KarMMa-RW study was defined as the date when patients became refractory to their last regimen. Baseline measurements for the real-world patients were collected after baseline and within 3 months of the patient becoming eligible, but prior to the start of the new regimen.

^b^Across all target doses.

^c^Baseline measurements for the KarMMa cohort were collected prior to the start of ide-cel infusion. Between screening and baseline assessment, ECOG performance scores deteriorated to 2 in 3 patients in KarMMa.

^d^Derived ISS was calculated using baseline values of albumin and beta-2-macroglobulin.

^e^Not collected or reported was defined as not collected, not reported, missing, or unknown.

The median age was 61 years (range, 33–78) and 64 years (range, 35–91), the median time from initial diagnosis was 6.0 years (range, 1.0–17.9) and 4.2 years (range, 0.4–17.7), and the median number of prior antimyeloma regimens was 6.0 (range, 3.0–16.0) and 5.0 (range, 3.0–12.0) in the KarMMa cohort and the Eligible RRMM cohort, respectively. Patients in the KarMMa cohort were more heavily pretreated than those in the Eligible RRMM cohort with 38.3% versus 19.5% having received ≥7 prior regimens. Additionally, more patients in the KarMMa cohort were double-class refractory (89.1% vs 53.7%) and triple-class refractory (84.4% vs 43.2%) (Table 1). The differences observed in disease characteristics between the Eligible RRMM and KarMMa cohorts were statistically adjusted. As shown in Supplementary Table 1, trimmed stabilized IPTW or matching based on propensity scores improved the balance of demographic features and patient characteristics across cohorts. After balancing using trimmed stabilized IPTW, the absolute standardized mean difference was <0.2 for each of the covariates with the exception of age and corrected calcium; however, these two unbalanced covariates were further adjusted and well balanced in the matching analysis. For the matched cohorts utilizing greedy nearest neighbor matching, all baseline covariates were well balanced with standardized mean differences of <0.09 for all covariates (Supplementary Table 2).

In the Eligible RRMM cohort, patients received 94 different treatment regimens as their subsequent (index) line of therapy (Supplementary Table 3). The three most common regimens were carfilzomib-pomalidomide-dexamethasone (8.4%), elotuzumab-lenalidomide-dexamethasone (5.3%), and carfilzomib-cyclophosphamide-dexamethasone (4.7%). All other regimens were used in ≤2.6% of patients in this cohort.

### Overall response rate

The efficacy parameters were significantly improved in the KarMMa cohort of all ide-cel treated patients across all target doses, compared with the Eligible RRMM cohort (Table 2). The ORR was 76.4% in the KarMMa cohort, versus 32.2% in the Eligible RRMM cohort (risk ratio [RR], 2.4 [95% CI, 1.7–3.3]; *P* < 0.0001). The rate of ≥VGPR was 57.9% in the KarMMa cohort, compared with 13.7% in Eligible RRMM patients (RR, 4.2 [95% CI, 2.5–7.2]; *P* < 0.0001). Improvements with ide-cel were greater when outcomes in KarMMa patients who received the highest target dose of 450 × 10^6^ CAR + T cells were compared with patients in the Eligible RRMM cohort. The ORR was 82.0% in the KarMMa cohort treated with a target dose of 450 × 10^6^ CAR + T cells versus 31.4% in the Eligible RRMM patients (RR, 2.6 [95% CI, 2.0–3.5]; *P* < 0.0001). The rate of ≥VGPR was 67.4% in the KarMMa patients treated with a target dose of 450 × 10^6^ CAR + T cells compared with 13.5% in the Eligible RRMM patients (RR, 5.0 [95% CI, 3.1–8.0]; *P* < 0.0001). Overall response rates adjusted for matching were consistent with the primary analysis, with an ORR of 71.6% in the Matched KarMMa cohort across all target doses versus 29.4% in the Matched RRMM cohort (RR, 2.4 [95% CI, 1.7–3.6]; *P* < 0.0001; Table 3).

**Table 2:** Response rates adjusted for stabilized trimmed inverse probability treatment weighting.

Response^a^	KarMMa cohort^b^ (*N* = 128)	Eligible RRMM cohort (*N* = 190)	KarMMa cohort 450 × 10^6^ CAR + T Cells (*N* = 54)	Eligible RRMM cohort (*N* = 190)
ORR (95% CI), %	76.4 (67.8–86.1)	32.2 (24.4–42.3)	82.0 (70.3–95.7)	31.4 (25.0–39.4)
RR (95% CI)	2.4 (1.7–3.3)		2.6 (2.0–3.5)	
* P*	<0.0001		<0.0001	
≥VGPR^**c**^ rate (95% CI), %	57.9 (47.8–70.1)	13.7 (8.6–21.9)	67.4 (52.6–86.4)	13.5 (9.1–20.1)
RR (95% CI)	4.2 (2.5–7.2)		5.0 (3.1–8.0)	
* P*	<0.0001		<0.0001	

ORR was defined as percentage of patients who achieved a best response of partial response or better.

≥VGPR rate was defined as percentage of patients who achieved a best response of VGPR or better.

*CI* confidence interval, *IPTW* inverse probability treatment weighting, *ORR* overall response rate, *RR* risk ratio, *VGPR* very good partial response.

^a^Derived for the KarMMa and Eligible RRMM cohorts using trimmed stabilized inverse probability treatment weighting propensity score.

^b^Across all target doses.

^c^Complete response not reported due to missing biopsy data in the Eligible RRMM cohort to confirm response.

**Table 3:** Overall response rates adjusted for matching.

Response^a^	Matched KarMMa^b^ (*N* = 76–80^c^)	Matched Eligible RRMM cohort (*N* = 76–80^c^)
ORR (95% CI), %	71.6 (61.5–83.3)	29.4 (20.2–42.8)
RR (95% CI)	2.4 (1.7–3.6)	
* P*	<0.0001	

*ORR* overall response rate, *RR* risk ratio.

^a^Derived for Matched Eligible RRMM and Matched KarMMa cohorts using greedy nearest neighbor matching with a caliper of 0.2 standard deviation of logit of the propensity score.

^b^Across all target doses.

^c^Number (range) of matched subjects from 30 imputed datasets; greedy nearest neighbor matching with a caliper.

### Progression-free survival and overall survival

**Fig. 2:**
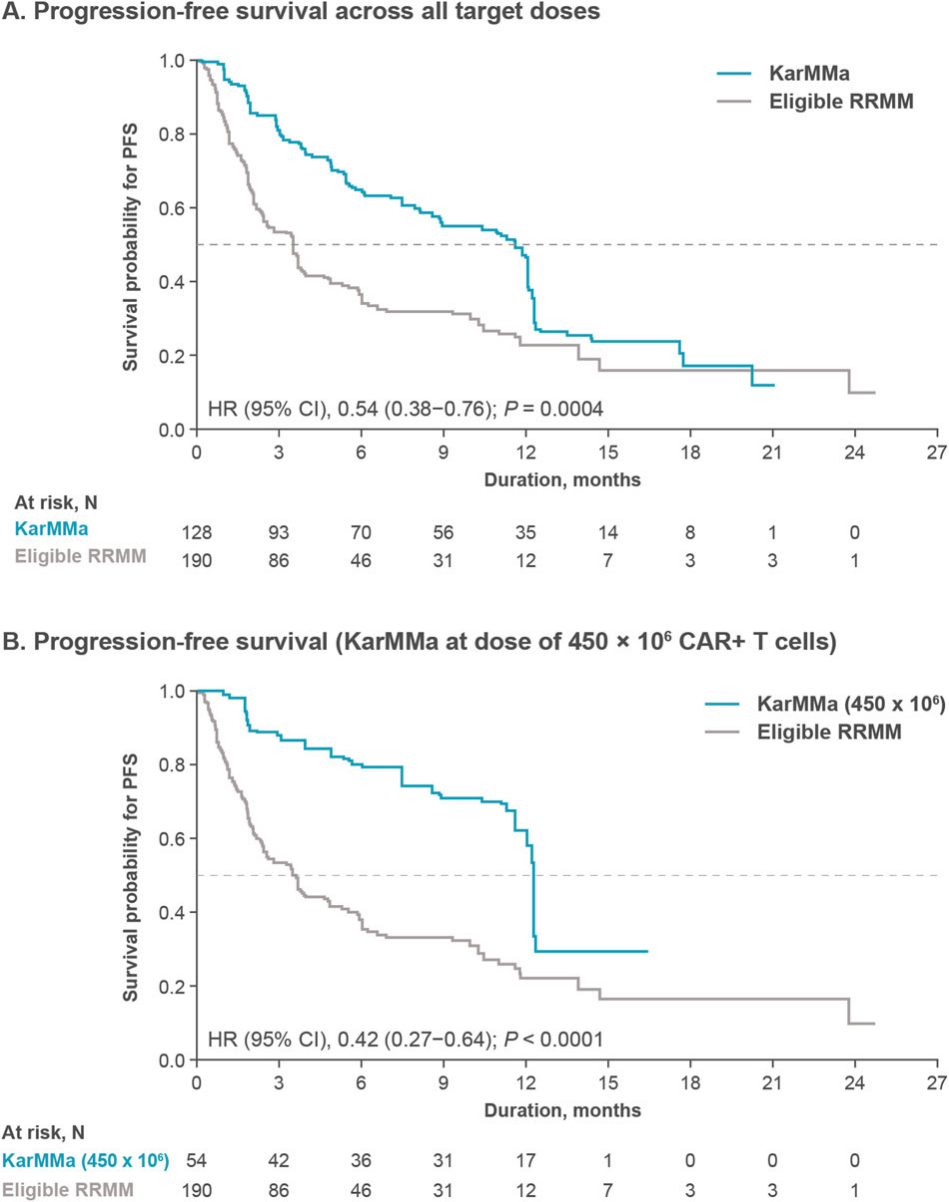
Progression-free survival. **A** shows a Kaplan–Meier curve of progression-free survival across all target doses and **B** shows a Kaplan–Meier curve of progression-free survival at the target dose of 450 × 10^6^ CAR + T cells based on Independent Response Committee Review according to IMWG criteria applying Food and Drug Administration censoring rules. IMWG denotes International Myeloma Working Group, NE not estimable.

Median PFS was significantly prolonged in KarMMa patients across all target doses, compared with Eligible RRMM patients (11.6 months vs 3.5 months; HR, 0.54 [95% CI, 0.38–0.76]; *P* = 0.0004); median follow-up was 12.9 months (range, 0.2–21.2) and 11.1 months (range, 0.2–24.0), respectively (Fig. 2A). Median PFS was 12.3 months at the highest target dose of 450 × 10^6^ CAR + T cells in the KarMMa cohort versus 3.5 months in the Eligible RRMM cohort (HR, 0.42 [95% CI, 0.27–0.64]; *P* < 0.0001) (Fig. 2B).

**Fig. 3:**
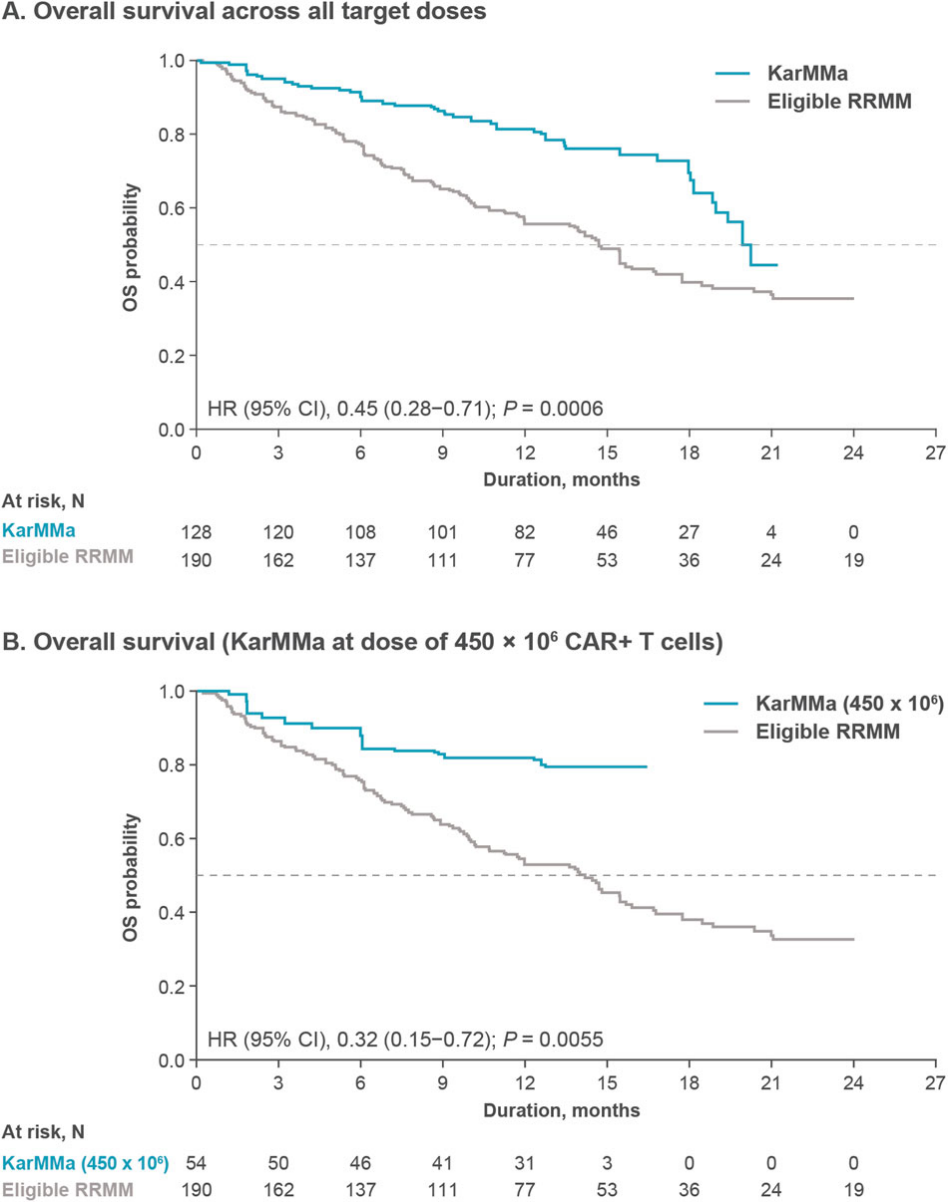
Overall survival. **A** shows a Kaplan–Meier curve of overall survival across all target doses and **B** shows a Kaplan–Meier curve of overall survival at the target dose of 450 × 10^6^ CAR + T cells based on Independent Response Committee Review according to IMWG criteria applying Food and Drug Administration censoring rules. IMWG denotes International Myeloma Working Group, NE not estimable.

**Fig. 4:**
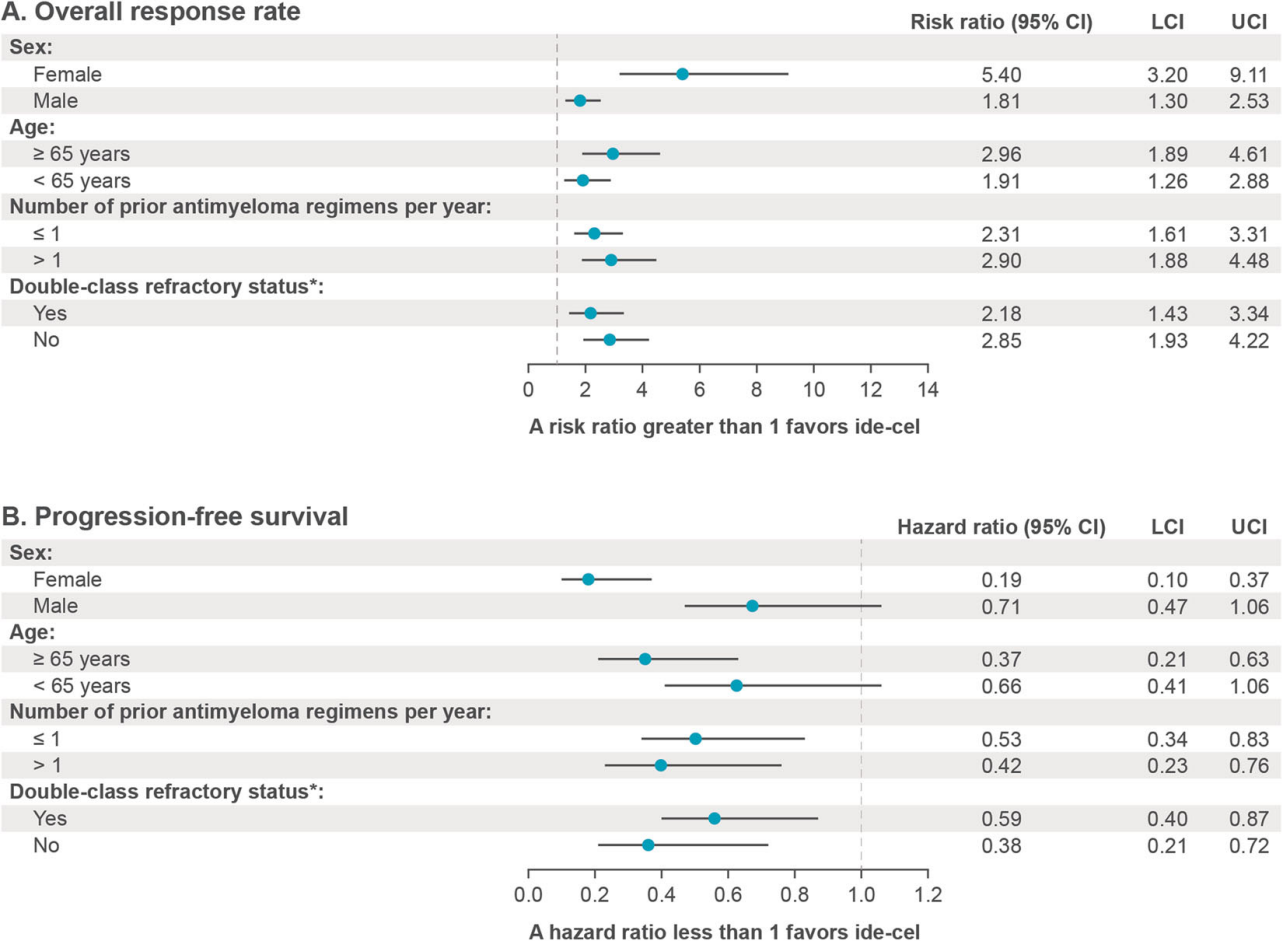
Subgroup analyses of overall response rate and progression-free survival. **A** shows overall response rate (confirmed partial response or better) by patient demographics and disease characteristics. **B** shows progression-free survival by patient demographics and disease characteristics. Patients with a partial response or better according to International Myeloma Working Group criteria applying Food and Drug Administration censoring rules were included. Multiple imputations were performed to create 30 datasets; estimates for the analyses were then obtained using Rubin’s rule to combine the individual estimates from each dataset. *Double-class refractory was defined as refractory to an immunomodulatory agent and a proteasome inhibitor. ORR denotes overall response rate, PFS progression-free survival.

Median OS was significantly improved with ide-cel in KarMMa across all target doses, versus the Eligible RRMM cohort (20.2 months vs 14.7 months; HR, 0.45 [95% CI, 0.28–0.71]; *P* = 0.0006); median follow-up among surviving patients was 14.4 months in the KarMMa cohort and 15.0 months in the Eligible RRMM cohort (Fig. 3A). Median OS was not reached at the highest target dose in the KarMMa cohort, versus 14.2 months in the Eligible RRMM cohort (HR, 0.32 [95% CI, 0.15–0.72]; *P* = 0.0055) (Fig. 3B). The estimated 12-month probability of surviving was 80% in the KarMMa cohort across all target doses and 56% in the Eligible RRMM cohort. The estimated 12-month probability of surviving was 82% at the target dose of 450 × 10^6^ CAR + T cells in the KarMMa cohort and 53% in the Eligible RRMM cohort. Furthermore, consistent improvements in ORR and PFS in the KarMMa cohort versus the Eligible RRMM cohort were demonstrated across all subgroups, including patients aged ≥65 years, patients with double-class refractory disease, and those with >1 prior antimyeloma regimen per year (Fig. 4).

### Duration of response and time to response

A numerical difference in the median DOR was observed between the KarMMa cohort across all target doses and the Eligible RRMM cohort (HR, 0.62 [95% CI, 0.33–1.16]; *P* = 0.1372) (supplementary Table 4). The median DOR was 11.1 months (95% CI, 10.8–11.5) in the KarMMa cohort and 9.0 months (95% CI, 7.5–10.5) in the Eligible RRMM cohort. The median TTR for responders in the KarMMa cohort was similar to the Eligible RRMM cohort (1.0 month [range, 0.5–8.8] and 1.1 months [range, 0.2–8.6], respectively).

## Discussion

Results from KarMMa-RW, a retrospective study of patients with triple-class exposed RRMM, confirmed that there is no standard of care therapy in this difficult-to-treat patient population. Real-world patients analyzed in this study received 94 different treatment regimens as a subsequent (index) line therapy, most commonly triplet combinations of immunotherapy, PIs or chemotherapy, and dexamethasone. Consistent with previous studies, demonstrating a poor prognosis in triple-class exposed RRMM patients [3, 16], clinical outcomes were suboptimal in the real-world RRMM patients, with an ORR of 32.2% and a median PFS of 3.5 months. With no clear standard of care, a more precise understanding of the expected outcomes with available therapies in RRMM in the real-world is needed.

In real-world RRMM patients, comprehensive large-scale data are limited. A comparative analysis of results from the STORM part 2 study in patients with triple-class exposed RRMM who received selinexor plus low-dose dexamethasone and a retrospective cohort from the Flatiron Health Analytic Database was conducted; however, the analysis had a limited sample size of <70 patients per group [34, 35]. The KarMMa-RW study is a large-scale, patient-level examination of outcomes with currently available treatments in real-world RRMM patients and the first study to directly compare results in triple-class exposed RRMM patients receiving ide-cel in the KarMMa study with outcomes in a similar group of real-world patients.

In KarMMa-RW, responses and survival outcomes were significantly improved in ide-cel−treated patients in the KarMMa study versus real-world RRMM patients (ORR and ≥VGPR, *P* < 0.0001; PFS, *P* = 0.0004; OS, *P* = 0.0006). Of note, the OS data were considered immature at the time of analysis. In addition, patients in the KarMMa study were more heavily pretreated and more had double- or triple-class refractory disease than real-world RRMM patients. A trend toward improved response durability was observed in favor of ide-cel (*P* = 0.1372), which may be explained by the smaller sample sizes of the compared cohorts due to the inclusion of only patients with a ≥PR (KarMMa cohort, *n* = 94; Eligible RRMM cohort, *n* = 58). Furthermore, consistent improvements in depth of response and survival were observed with ide-cel across subgroups, including older patients, patients with double-class refractory disease, and those who received multiple prior regimens per year. As the ongoing KarMMa study demonstrated greatest efficacy at the highest target dose of 450 × 10^6^ CAR + T cells (ORR, 82%; median DOR, 11.3 months; median PFS, 12.1 months) [25], outcomes in real-world RRMM patients were compared with those observed in KarMMa patients who received 450 × 10^6^ CAR + T cells. Results were consistent with the comparison across ide-cel dose levels, with significant improvements in response rates (ORR and ≥VGPR, *P* < 0.0001) and survival (PFS; *P* < 0.0001, OS; *P* = 0.0055) observed with ide-cel. Given the small sample size at the highest target dose, a comparison of DOR and TTR was not feasible.

Patients with RRMM who progress on anti-CD38 antibody therapy have poor survival outcomes, as demonstrated in the retrospective MAMMOTH study, with a median PFS of 3.4 months and a median OS of 9.3 months [16]. Results are similar with recently approved therapies; selinexor plus dexamethasone treatment resulted in a median PFS of 3.7 months and median OS of 8.6 months in the STORM part 2 study and belantamab mafodotin resulted in a median PFS of 2.8 months (2.5 mg/kg) and 3.9 months (3.4 mg/kg) [36, 37]. To compare outcomes with selinexor plus dexamethasone or belantamab mafodotin with those of a one-time infusion of ide-cel in triple-class exposed RRMM, a matching adjusted indirect comparison was performed which demonstrated improvements in ORR, PFS, and OS in ide-cel–treated patients [18, 38].

In KarMMa-RW, the stringent inclusion and exclusion criteria applied to select patients with comparable baseline features as well as the propensity score methods ensured robust and reliable comparisons with the KarMMa study population. Sensitivity analyses, which included only patients with an Eastern Cooperative Oncology Group performance status of 0 or 1 within the real-world Eligible RRMM cohort, confirmed the overall results. However, rigorous selection criteria for matching resulted in only 76–80 patients in the Eligible RRMM and KarMMa cohorts. Thus, the exact number of patients matched varied for the 30 different datasets generated through multiple imputations. Throughout the study, trimmed stabilized IPTW was applied to optimize the number of adjusted and compared patients in the Eligible RRMM and KarMMa cohorts. All covariates were well balanced for the trimmed IPTW analysis except age and calcium, which were further balanced after matching. As the results were presented with trimmed stabilized IPTW to account for differences in patient characteristics between the two cohorts, outcomes from KarMMa differ slightly from previously reported outcomes in the primary KarMMa study [25].

Limitations of this study include potential bias and the fact that unmeasured confounders could not be controlled, which may have influenced the balancing of the two cohorts. Additionally, since this study was designed to develop a synthetic cohort reflective of the KarMMa population and excluded patients not healthy enough to receive ide-cel treatment as a next line of therapy, the 190 real-world Eligible RRMM patients selected from the broad RRMM cohort (*N* = 1949) may not fully represent the general RRMM population. For this reason, clinical implications of the improved outcomes observed with ide-cel therapy should be carefully drawn, accounting for a patient’s health status and treatment history in real-world practice settings.

Response rate, depth of response, and time to disease progression decrease with each subsequent line of therapy, making late-stage multiple myeloma difficult to treat effectively [15, 39–41]. As confirmed by the KarMMa-RW study, outcomes are suboptimal with current treatment options in real-world RRMM patients who have received three or more prior therapies, including an immunomodulatory agent, a PI, and an anti-CD38 antibody. This study demonstrated a clear benefit with ide-cel treatment over currently available therapies, with significant increases in efficacy. Thus, ide-cel offers a promising new treatment option in triple-class exposed RRMM.

## Data sharing statement

Bristol-Myers Squibb Company policy on data sharing may be found at https://www.bms.com/researchers-and-partners/independent-research/data-sharing-request-process.html.

## Supplementary information

Supplementary Information

## References

[1] Ferlay J, Colombet M, Soerjomataram I, Mathers C, Parkin DM, Piñeros M (2019). Estimating the global cancer incidence and mortality in 2018: Globocan sources and methods. Int J Cancer.

[2] Rajkumar SV, Kumar S (2016). Multiple myeloma: diagnosis and treatment. Mayo Clin Proc.

[3] Nijhof IS, van de Donk NWCJ, Zweegman S, Lokhorst HM (2018). Current and new therapeutic strategies for relapsed and refractory multiple myeloma: an update. Drugs.

[4] Kumar SK, Dispenzieri A, Lacy MQ, Gertz MA, Buadi FK, Pandey S (2014). Continued improvement in survival in multiple myeloma: changes in early mortality and outcomes in older patients. Leukemia.

[5] Jagannath S, Rifkin RM, Gasparetto CJ, Toomey K, Durie B, Hardin JW (2020). Treatment journeys of patients with newly diagnosed multiple myeloma (NDMM): results from the connect MM registry. Clin Lymphoma Myeloma Leuk.

[6] Darzalex® (daratumumab) [package insert]. Horsham, PA: Janssen Biotech, Inc; 2020.

[7] Sarclisa® (isatuximab-irfc) [package insert]. Bridgewater, NJ: Sanofi-Aventis U.S. LLC; 2020.

[8] Lokhorst HM, Plesner T, Laubach JP, Nahi H, Gimsing P, Hansson M (2015). Targeting CD38 with daratumumab monotherapy in multiple myeloma. N Engl J Med.

[9] Baker H (2016). Daratumumab improves survival in multiple myeloma. Lancet Oncol.

[10] Empliciti® (elotuzumab) [package insert]. Princeston, NJ: Bristol-Myers Squibb Company; 2018.

[11] Xpovio® (selinexor) [package insert]. Newton, MA; Karyopharm Therapeutics Inc; 2020.

[12] Attal M, Richardson PG, Rajkumar SV, San-Miguel J, Beksac M, Spicka I (2019). Isatuximab plus pomalidomide and low-dose dexamethasone versus pomalidomide and low-dose dexamethasone in patients with relapsed and refractory multiple myeloma (ICARIA-MM): A randomised, multicentre, open-label, phase 3 study. Lancet.

[13] Farydak® (panobinostat) [package insert]. Las Vegas, NV: Secura, Inc; 2015.

[14] Harvey RD (2014). Incidence and management of adverse events in patients with relapsed and/or refractory multiple myeloma receiving single-agent carfilzomib. Clin Pharmacol.

[15] Kumar SK, Dimopoulos MA, Kastritis E, Terpos E, Nahi H, Goldschmidt H (2017). Natural history of relapsed myeloma, refractory to immunomodulatory drugs and proteasome inhibitors: A multicenter IMWG study. Leukemia.

[16] Gandhi UH, Cornell RF, Lakshman A, Gahvari ZJ, McGehee E, Jagosky MH (2019). Outcomes of patients with multiple myeloma refractory to CD38-targeted monoclonal antibody therapy. Leukemia.

[17] Mutiple myeloma (version 2.2021). [Available from: https://www.nccn.org/professionals/physician_gls/pdf/myeloma.pdf] Accessed 29 September 2020.

[18] Blenrep®(belantamab mafodotin-blmf) [package insert]. Middlesex, UK: Glaxosmithkline; 2020.

[19] Mikhael J (2020). Treatment options for triple-class refractory multiple myeloma. Clin Lymphoma Myeloma Leuk.

[20] Fulciniti M, Munshi NC, Martinez-Lopez J (2015). Deep response in multiple myeloma: a critical review. Biomed Res Int.

[21] Telford C, Kabadi SM, Abhyankar S, Song J, Signorovitch J, Zhao J (2019). Matching-adjusted indirect comparisons of the efficacy and safety of acalabrutinib versus other targeted therapies in relapsed/refractory mantle cell lymphoma. Clin Ther.

[22] Cornell C, Hari P, Tang S, Biran N, Callander N, Chari A, et al. Overall survival of patients with triple-class refractory multiple myeloma treatedwith selinexor Q12 plus dexamethasone versus standard of care in MAMMOTH. Am J Hematol. 2021;96:E5–E8. 10.1002/ajh.26010 32974944

[23] Friedman KM, Garrett TE, Evans JW, Horton HM, Latimer HJ, Seidel SL (2018). Effective targeting of multiple B-cell maturation antigen-expressing hematological malignances by anti-B-cell maturation antigen chimeric antigen receptor T cells. Hum Gene Ther.

[24] Lin Y, Raje NS, Berdeja JG, Siegel DS, Jagannath S, Madduri D, et al. Idecabtagene vicleucel (ide-cel, bb2121), a BCMA-directed CAR T cell therapy, in patients with relapsed and refractory multiple myeloma: Updated results from phase 1 CRB-401 study. American Society of Hematology (ASH). Abstract 131 (2020).

[25] Munshi NC, Anderson LD JR, Shah N, Madduri D, Berdeja J, Lonial S (2021). Idecabtagene vicleucel in relapsed and refractory multiple myeloma. N Engl J Med.

[26] Crump M, Neelapu SS, Farooq U, Van Den Neste E, Kuruvilla J, Westin J (2017). Outcomes in refractory diffuse large B-cell lymphoma: results from the international SCHOLAR-1 study. Blood.

[27] Chen C, Armand P, Rogula B, Johnston K, Peterson D, Connors JM (2019). A matching-adjusted indirect comparison of nivolumab versus brentuximab vedotin for relapsed/refractory classical Hodgkin lymphoma after failure of autologous hematopoietic cell transplantation. Blood.

[28] Kumar S, Paiva B, Anderson KC, Durie B, Landgren O, Moreau P (2016). International myeloma working group consensus criteria for response and minimal residual disease assessment in multiple myeloma. Lancet Oncol.

[29] Ailawadhi S, Jagannath S, Narang M, Rifkin RM, Terebelo HR, Toomey K (2020). Connect MM registry as a national reference for united states multiple myeloma patients. Cancer Med.

[30] Austin PC (2011). An introduction to propensity score methods for reducing the effects of confounding in observational studies. Multivariate Behav Res.

[31] Austin PC, Stuart EA (2015). Moving towards best practice when using inverse probability of treatment weighting (IPTW) using the propensity score to estimate causal treatment effects in observational studies. Stat Med.

[32] Delarue R, Tilly H, Mounier N, Petrella T, Salles G, Thieblemont C (2013). Dose-dense rituximab-chop compared with standard rituximab-chop in elderly patients with diffuse large B-cell lymphoma (the LNH03-6B study): A randomised phase 3 trial. Lancet Oncol.

[33] Raje N, Berdeja J, Lin Y, Siegel D, Jagannath S, Madduri D (2019). Anti-BCMA CAR T-cell therapy bb2121 in relapsed or refractory multiple myeloma. N Engl J Med.

[34] Richardson PG, Jagannath S, Chari A, Vogl DT, Dimopoulos MA, Moreau P (2019). Overall survival (OS) with oral selinexor plus low dose dexamethasone (Sd) in patients with triple class refractory-multiple myeloma (TCR-MM). J Clin Oncol.

[35] Jung PS, Kim DY, Lee SW, Park JY, Suh DS, Kim JH (2015). Clinical role of adjuvant chemotherapy after radical hysterectomy for FIGO stage IB-IIA cervical cancer: comparison with adjuvant RT/CCRT using inverse-probability-of-treatment weighting. PLoS ONE.

[36] Chari A, Vogl DT, Gavriatopoulou M, Nooka AK, Yee AJ, Huff CA (2019). Oral selinexor-dexamethasone for triple-class refractory multiple myeloma. N Engl J Med.

[37] Lonial S, Lee HC, Badros A, Trudel S, Nooka AK, Chari A, et al. DREAMM-2: single-agent belantamab mafodotin in relapsed/refractory multiple myeloma refractory to proteosome inhibitors, immunomodulatory agents, and refractory and/or intolerant to anti-CD38 mAbs. European Hematology Association (EHA) Abstract. EP970, 2020.

[38] Rodriguez-Otero P, Weisel K, Davies F, Delforge M, Ayers D, Cope S, et al. Matching-adjusted indirect comparisons of efficacy outcomes for idecabtagene vicleucel from the KarMMa study vs selinexor plus dexamethasone (STORM part 2) and belantamab mafodotin (DREAMM-2). European Hematology Association (EHA) Presentation EP969, 2020.

[39] Yong K, Delforge M, Driessen C, Fink L, Flinois A, Gonzalez-McQuire S (2016). Multiple myeloma: patient outcomes in real-world practice. Br J Haematol.

[40] Lonial S, Weiss BM, Usmani SZ, Singhal S, Chari A, Bahlis NJ (2016). Daratumumab monotherapy in patients with treatment-refractory multiple myeloma (SIRIUS): an open-label, randomised, phase 2 trial. Lancet.

[41] Usmani SZ, Weiss BM, Plesner T, Bahlis NJ, Belch A, Lonial S (2016). Clinical efficacy of daratumumab monotherapy in patients with heavily pretreated relapsed or refractory multiple myeloma. Blood.

