# Supplementary material for: KarMMa-RW: comparison of idecabtagene vicleucel with real-world outcomes in relapsed and refractory multiple myeloma
Source: Blood Cancer J. 2021 Jun 18;11(6):116. doi: 10.1038/s41408-021-00507-2 (PMC8213772; doi:10.1038/s41408-021-00507-2)
Supplement: Supplementary file 1 — Supplementary Information [file 41408_2021_507_MOESM1_ESM.docx]

**Supplementary Information**

**KarMMa-RW: Comparison of idecabtagene vicleucel with real-world outcomes in relapsed and refractory multiple myeloma**

Sundar Jagannath, Yi Lin, Hartmut Goldschmidt, Donna Reece, Ajay Nooka, Alicia Senin, Paula Rodriguez Otero, Ray Powles, Kosei Matsue, Nina Shah, Larry D. Anderson, Jr, Matthew Streetly, Kimberly Wilson, Hoa Van Le, Arlene S. Swern, Amit Agarwal, and David S. Siegel

**Supplementary Methods**

***Study design and patients***

In the Eligible RRMM cohort, patients had to be refractory to their last and have measurable disease by monoclonal-protein and/or serum free light chain levels. Refractory to a last regimen was defined as documented disease progression during or within 60 days of completing the last regimen before study entry. Measurable disease was defined as monoclonal-protein levels of ≥1.0 g/dL in serum and ≥200 mg/24 h in urine, and/or serum free light chain levels of ≥10 mg/dL, provided serum free light chain ratio is abnormal.

Exclusion criteria included Eastern Cooperative Oncology Group performance status ≥2 at the date that the real-world patient became refractory to the last regimen (baseline), central nervous system involvement with multiple myeloma; history or presence of clinically relevant central nervous system pathology, solitary plasmacytomas without other evidence of measurable disease, active or history of plasma cell leukemia, secondary malignancies requiring therapy in the last 3 years or that are not in complete remission.

***Data sources***

Acquisition of clinical site data was managed by IQVIA (Human Data Science Company™) and obtained through manual chart abstraction into the study electronic case report form. The external databases included COTA real-world evidence database, Flatiron, Guardian Research Network, and M2Gen. COTA generates longitudinal patient records with detailed clinical, demographic, testing, treatment, and outcome information from both structured and unstructured fields. The Flatiron Health database is a longitudinal, demographically, and geographically diverse database consisting of de-identified patient-level data derived from structured electronic health records, unstructured reports, and physician notes collected from chart abstraction. The Guardian Research Network is a nationwide consortium of regional community health systems harnessing real-time clinical and molecular oncology de-identified patient electronic health records information. M2Gen is an alliance of 19 of the nation’s leading cancer centers that partners with ORIEN™ network; collection of longitudinal clinical data was allowed from patients who receives care at one of the ORIEN™ cancer centers.

***Propensity score balancing***

To ensure a balance of baseline characteristics, propensity score trimmed stabilized inverse probability treatment weighting (IPTW) was used to weight the real-world cohorts and the respective KarMMa cohorts. The following steps were carried out separately for each of the populations. As a sensitivity analysis, propensity score balancing was carried out for the treated patients.

*Step 1: Selection of covariates to include in multivariate logistic regression modeling*

Variables for consideration in the propensity score analysis were selected based on clinical importance relating to outcome and disease severity based on the expertise of the study team with a further review by a team of experts. An expert panel consisting of 7 clinical experts selected and ranked the order of prognostic factors across studies using a structured approach. Univariate logistic regression was also used to identify potential covariates for the initial propensity score model. Covariates considered included age, sex, bone lesions, time from initial diagnosis, number of prior regimens, cytogenetic risk (high/low), refractoriness to immunomodulatory agents, proteasome inhibitors (PIs), or anti-CD38 antibodies, and baseline laboratory tests (platelet, hemoglobin, albumin, and calcium). The analysis allowed for as much as 30% missing data for highly prognostic covariates in the Eligible RRMM cohort. Any values missing for the candidate covariates were imputed via multiple imputation procedures using the full conditional specification statement SAS procedure (PROC MI).

*Step 2: Propensity score modeling*

Variables identified as statistically significant (*P*≤0.15) in the model were included in the initial/full multivariate logistic regression. All possible models from the full model were evaluated with the best model fit being selected to obtain a propensity score. The final propensity score model included age; baseline albumin; number of prior regimens; number of prior regimens per year; creatinine clearance group; prior refractoriness to immunomodulatory agents, PIs, and anti-CD38 antibodies; bone lesions; and baseline calcium.

The final propensity score obtained for each imputed data set and the propensity score stabilized IPTW were then used to perform the balancing. For the clinical trial, the weights were 1/propensity score times the proportion of subjects from the clinical trial. For the real-world study, the weights are 1/(1−propensity score) times the proportion of real-world patients. The propensity score model that achieved the best balance was chosen as the primary model.

*Step 3: Assessment of balance between cohorts on using propensity scores*

Assessment of balance in propensity score between cohorts was conducted through a side-by-side comparison of raw baseline data for the Eligible RRMM cohort and the KarMMa cohort and raw and inverse propensity score weighted Eligible RRMM and KarMMa baseline data. For these comparisons, pooled standardized mean differences were computed using Rubin’s rules before and after balancing. For the pooled standardized differences, a threshold of 0.2 was used to indicate potentially important imbalances.^1^

*Step 4: Balancing methods and criteria*

It was originally planned to provide 2:1 match for the Eligible RRMM cohort and the KarMMa cohort; however, it actually included the same number of real-world patients as in the KarMMa cohort. The IPTW was chosen as the primary methodology with a sensitivity analysis using the greedy nearest neighbor matching (with caliper width of 0.2 standard deviation of the logit of propensity score).

The greedy nearest neighbor match took the closest neighbor real-world patient in the Eligible RRMM cohort, matched to the patient in the KarMMa study. This matching was done without replacement so that once a pair was matched, they were removed from the pool of candidates for matching. In this 1:1 greedy nearest neighbor matching one patient in the real-world cohort was matched with a single patient in the KarMMa study, such that the matching produced the smallest within-pair difference among all available pairs with this treated unit. Matching was done based on the logit of the propensity using a caliper distance of 0.2 standard deviation.  A dataset of propensity score was provided by patient, matched set, and cohort prior to conducting any analyses.

*Step 5: Risk ratios and hazards ratios*

Risk ratios and hazards ratios were then estimated for each of the 30 datasets. Overall estimates were then obtained using Rubin’s rules. This approach provided unbiased estimates with appropriate confidence intervals.

*Step 6: Analysis firewall*

For the initial wave of analysis, there was a firewall between the propensity score balancing process and the analysis of the postbaseline response and outcome data. The balancing process was conducted by statisticians and programmers without knowledge of outcome data. Once the IPTW and the matching flags were generated and frozen, the statisticians and programmers were given access to the outcome data.

**Supplementary Table 1.** **Balance of baseline demographics and disease characteristics**

|  | **Before balancing** | | | **After balancing** | | |
| --- | --- | --- | --- | --- | --- | --- |
| **Covariate*** | **KarMMa cohort^†^**  **(N=128)** | **Eligible RRMM cohort**  **(N=190)** | **SMD**  **(KarMMa − Eligible RRMM)** | **KarMMa cohort^†^**  **(N=128)** | **Eligible RRMM cohort**  **(N=190)** | **SMD (KarMMa − Eligible RRMM)** |
| **Age, years** | 59.8 | 64.5 | −0.5068 | 60.8 | 62.9 | −0.2189 |
| **Male, %** | 60.0 | 60.0 | 0.0194 | 60.0 | 60.0 | −0.0753 |
| **Time since initial diagnosis** | 6.9 | 4.9 | 0.5814 | 6.3 | 5.9 | 0.1289 |
| **Corrected calcium, mmol/L** | 3.0 | 2.4 | 0.4302 | 2.8 | 2.5 | 0.2693 |
| **Number of prior regimens** | 5.6 | 4.8 | 0.5288 | 5.1 | 5.1 | 0.0611 |
| **Number of prior regimens per year since diagnosis** | 1.2 | 1.3 | −0.1423 | 1.1 | 1.2 | −0.0770 |
| **Triple-class refractory status,^‡^ %** | 80.0 | 40.0 | 0.9491 | 60.0 | 60.0 | 0.1268 |

SMD, standardized mean difference.

Multiple imputation procedures created 30 datasets and overall estimates were obtained using Rubin’s rules to combine the individual estimates. The stabilized inverse probability treatment weighting was trimmed at the maximum of the minimum weight and minimum of the maximum weight for the KarMMa cohort and the Eligible RRMM cohort.

*A covariate was not included in the balancing if it had > 30% missing for the Eligible RRMM cohort. Means are presented for continuous variables and proportions are presented for categorical variables. Standardized mean difference was obtained from the KarMMa cohort minus the Eligible RRMM cohort and used trimmed stabilized weights when combining the mean and standard deviation.

^†^Across all target doses.

**^‡^**Triple-class refractory was defined as refractory to an immunomodulatory agent, a proteasome inhibitor, and an anti-CD38 antibody.

**Supplementary Table 2. Balance of baseline demographics and disease characteristics before and after matching**

|  | **Before matching** | | | **After matching** | | |
| --- | --- | --- | --- | --- | --- | --- |
| **Covariate*** | **KarMMa Cohort^†^**  **(N=128)** | **Eligible RRMM Cohort**  **(N=190)** | **SMD (KarMMa-Eligible RRMM)** | **KarMMa Cohort^†^**  **(N=76–80)** | **Eligible RRMM Cohort**  **(N=76–80)** | **SMD (KarMMa − Eligible RRMM)** |
| **Age, years** | 59.8 | 64.5 | −0.5068 | 61.7 | 62.2 | −0.0520 |
| **Male, %** | 60.0 | 60.0 | 0.0194 | 60.0 | 60.0 | 0.0007 |
| **Corrected Calcium, mmol/L** | 3.0 | 2.4 | 0.4302 | 2.5 | 2.5 | 0.0003 |
| **Time since initial diagnosis** | 6.9 | 4.9 | 0.5814 | 5.8 | 5.6 | 0.0856 |
| **Number of prior regimens** | 5.6 | 4.8 | 0.5288 | 5.3 | 5.1 | 0.0851 |
| **Number of prior regimens per year since diagnosis** | 1.2 | 1.3 | −0.1423 | 1.2 | 1.2 | 0.0148 |
| **Triple-class refractory status,^‡^ %** | 80.0 | 40.0 | 0.9491 | 80.0 | 80.0 | 0.0165 |

SMD, standardized mean difference.

Multiple imputation procedures created 30 datasets and overall estimates were obtained using Rubin’s rules to combine the individual estimates. The stabilized inverse probability treatment weighting was trimmed at the maximum of the minimum weight and minimum of the maximum weight for the KarMMa cohort and the Eligible RRMM cohort.

*A covariate was not included in the balancing if it had > 30% missing for the Eligible RRMM cohort. Means are presented for continuous variables and proportions are presented for categorical variables. Standardized mean difference was obtained from the KarMMa cohort minus the Eligible RRMM cohort and used trimmed stabilized weights when combining the mean and standard deviation.

^†^Across all target doses.

**^‡^**Triple-class refractory was defined as refractory to an immunomodulatory agent, a proteasome inhibitor, and an anti-CD38 antibody.

**Supplementary Table 3. Most common anti-myeloma treatment regimens in ≥5 patients**

| **Treatment regimen** | **Eligible RRMM cohort (N=190)**  **n (%)** |
| --- | --- |
| **Carfilzomib-pomalidomide-dexamethasone** | 16 (8.4) |
| **Elotuzumab-lenalidomide-dexamethasone** | 10 (5.3) |
| **Carfilzomib-cyclophosphamide-dexamethasone** | 9 (4.7) |
| **Carfilzomib-dexamethasone** | 5 (2.6) |
| **Cisplatin-cyclophosphamide-dexamethasone-etoposide** | 5 (2.6) |
| **Daratumumab-dexamethasone-lenalidomide** | 5 (2.6) |
| **Daratumumab-dexamethasone-pomalidomide** | 5 (2.6) |
| **Dexamethasone-pomalidomide** | 5 (2.6) |

**Supplementary Table 4. Duration of and time to response adjusted for stabilized trimmed inverse probability treatment weighting**

| **Parameter** | **KarMMa cohort* (N=128)** | **Eligible RRMM cohort (N=190)** |
| --- | --- | --- |
| **Duration of response, months^†,‡^** |  |  |
| **Median (95% CI)^§^** | 11.1 (10.8-11.5) | 9.0 (7.5-10.5) |
| **HR (95% CI)^ǁ^** | 0.62 (0.33-1.16) | |
| ***P*^ǁ^** | 0.1372 | |
| **Responders, n (%)** | 94 (73.4) | 58 (30.5) |
| **Time to response, months^†,‡^** |  |  |
| **Median (range)** | 1.0 (0.5-8.8) | 1.1 (0.2-8.6) |

CI, confidence interval; HR, hazard ratio.

*Across all target doses.

^†^Time to response and duration of response analyses are based on responders only. For summary of time to response categories, a 3-day window is added to each month for Month 1 to Month 8; and a 14-day window is added to Month 9 and onwards.

^‡^Only subjects with a response of stringent complete response, complete response, very good partial response (VGPR), or partial response were included in the analysis.

^§^The median was based on the adjusted product-limit estimate with inverse probability treatment weighting trimmed at the maximum of the minimum weight and the minimum of the maximum weight for the ide-cel cohort and the Eligible RRMM cohort.

^ǁ^HR and CI were based on a Cox model with study (Eligible RRMM or ide-cel cohorts) as a term in the model and as using IPTWs trimmed at the maximum of the minimum weight and the minimum of the maximum weight for the KarMMa and Eligible RRMM cohorts, as well as adjusted for unbalanced covariates used in the propensity score model.

**Supplementary References**

1. Austin PC. Balance diagnostics for comparing the distribution of baseline covariates between treatment groups in propensity-score matched samples. *Stat Med*. 2009;28(25):3083-3107.
